# Calcitriol downregulates fibroblast growth factor receptor 1 through histone deacetylase activation in HL-1 atrial myocytes

**DOI:** 10.1186/s12929-018-0443-3

**Published:** 2018-05-18

**Authors:** Ting-Wei Lee, Ting-I Lee, Yung-Kuo Lin, Yu-Hsun Kao, Yi-Jen Chen

**Affiliations:** 1 Graduate Institute of Clinical Medicine, College of Medicine, Taipei Medical University, 250 Wu-Xing Street, Taipei, 11031 Taiwan; 2 Division of Endocrinology and Metabolism, Department of Internal Medicine, Wan Fang Hospital, Taipei Medical University, Taipei, Taiwan; 3 Department of General Medicine, School of Medicine, College of Medicine, Taipei Medical University, Taipei, Taiwan; 4 Division of Cardiovascular Medicine, Department of Internal Medicine, Wan Fang Hospital, Taipei Medical University, Taipei, Taiwan; 5 Division of Cardiology, Department of Internal Medicine, School of Medicine, College of Medicine, Taipei Medical University, Taipei, Taiwan; 6 Department of Medical Education and Research, Wan Fang Hospital, Taipei Medical University, Taipei, Taiwan

**Keywords:** Calcitriol, Cardiomyocyte, Fibroblast growth factor receptor, Histone deacetylase, Vitamin D

## Abstract

**Background:**

Fibroblast growth factor (FGF)-2 plays a crucial role in the pathophysiology of cardiovascular diseases (CVDs). FGF-2 was reported to induce cardiac hypertrophy through activation of FGF receptor 1 (FGFR1). Multiple laboratory findings indicate that calcitriol may be a potential treatment for CVDs. In this study, we attempted to investigate whether calcitriol regulates FGFR1 expression to modulate the effects of FGF-2 signaling in cardiac myocytes and explored the potential regulatory mechanism.

**Methods:**

Western blot, polymerase chain reaction, small interfering RNA, fluorometric activity assay, and chromatin immunoprecipitation (ChIP) analyses were used to evaluate FGFR1, FGFR2, FGFR3, FGFR4, phosphorylated extracellular signal-regulated kinase (p-ERK), β-myosin heavy chain (β-MHC), phosphorylated phospholipase Cγ (p-PLCγ), nuclear factor of activated T cells (NFAT), and histone deacetylase (HDAC) expressions and enzyme activities in HL-1 atrial myocytes without and with calcitriol (1 and 10 nM) treatment, in the absence and presence of FGF-2 (25 ng/mL) or suberanilohydroxamic acid (SAHA, a pan-HDAC inhibitor, 1 μM).

**Results:**

We found that calcitriol-treated HL-1 cells had significantly reduced FGFR1 expression compared to control cells. In contrast, expressions of FGFR2, FGFR3, and FGFR4 were similar between calcitriol-treated and control HL-1 cells. FGF-2-treated HL-1 cells had similar PLCγ phosphorylation and nuclear/cytoplasmic NFAT expressions compared to control cells. FGF-2 induced lower expressions of p-ERK and β-MHC in calcitriol-treated HL-1 cells than in control cells. FGFR1-knockdown blocked FGF-2 signaling and reversed the protective effects of calcitriol. Compared to control cells, calcitriol-treated HL-1 cells had higher nuclear HDAC activity. The ChIP analysis demonstrated a significant decrease in acetyl-histone H4, which is associated with an increase in HDAC3 in the FGFR1 promoter. Calcitriol-mediated FGFR1 downregulation was attenuated in the presence of SAHA.

**Conclusions:**

Calcitriol diminished FGFR1 expression through HDAC activation, which ameliorated the harmful effects of FGF-2 on cardiac myocytes.

## Background

Cardiovascular diseases (CVDs) are a major cause of morbidity and mortality worldwide. There is a growing body of evidence suggesting that fibroblast growth factor (FGF)-2 plays a crucial role in the pathophysiology of CVDs. FGF-2 is a signaling protein and is expressed by multiple cell types in the adult myocardium, including cardiac myocytes, fibroblasts, and smooth muscle cells [1]. FGF-2 is released from damaged cells and by an exocytotic mechanism [1, 2]. FGF-2-deficient mice developed significantly less cardiac hypertrophy in response to transverse aortic coarctation [3]. Studies with FGF-2-knockout and transgenic mice demonstrated that FGF-2 mediates the cardiac hypertrophic response to isoproterenol and angiotensin II through activation of extracellular signal-regulated kinase (ERK) signaling [4, 5]. Moreover, FGF-2 levels in a pericardial effusion were elevated in patients with inflammatory pericardial effusion, suggesting that FGF-2 participates in the pathogenesis of inflammatory pericardial disease [6]. As do other paracrine FGFs, FGF-2 exerts biological activities by binding to cell surface FGF receptors (FGFRs) [2, 7]. The mammalian genome encodes four FGFR isoforms, FGFR1~ 4, which belong to the receptor tyrosine kinase family [2, 8]. FGFR1 is the predominant receptor that transduces the cardiac effects of FGF-2 [1, 2, 7]. Expressions of different FGFR isoforms are tissue-specific. FGFR1 is predominantly expressed in the mouse heart [9]. Analysis with immunohistochemical (IHC) staining of normal human tissues also revealed marked expression of FGFR1 in cardiac myocytes [10]. Furthermore, a recent study showed that cardiomyocyte-specific overexpression of constitutively active FGFR1 in mice increased cardiac contractility and resulted in hypertrophic cardiomyopathy [11]. However, the regulatory mechanism of FGFR1 expression in the heart has not been fully elucidated.

Calcitriol, a bioactive metabolite of vitamin D, exerts multiple beneficial effects on the cardiovascular system [12–14], and epidemiological data showed that a vitamin D deficiency increases the risk of CVDs and heart failure [12, 15, 16]. Our previous studies showed that calcitriol may attenuate cardiac hypertrophy in diabetic animals [17, 18]. Since FGFR1 critically contributes to cardiac pathophysiology, calcitriol may regulate cardiac FGFR1, resulting in cardiovascular impacts. Histone deacetylases (HDACs) are a group of enzymes that regulate gene expressions by histone deacetylation. They remove acetyl groups from lysine residues present in histones and other proteins and negatively modulate gene transcription by making histones wrap DNA more tightly [19]. Recruitment of HDACs was implicated as a possible mechanism of vitamin D-induced transcriptional regulation [20]. In this study, we investigated the effects of calcitriol on FGFR1 in cardiac myocytes, and explored whether calcitriol modulates HDACs to regulate FGFR1 signaling.

## Methods

### Cell culture

HL-1 cells derived from mouse atrial cardiac muscle cells [21] (kindly provided by Dr. Claycomb, Louisiana State University Medical Center, New Orleans, LA) were cultured in a humidified atmosphere of 5% CO_2_ at 37 °C in Claycomb medium (JRH Biosciences, Lenexa, KS). Calcitriol (1 and 10 nM) was used to treat HL-1 cells for 48 h to determine the initial dose-response effect. Based on results of the dose-response relationship, 10 nM calcitriol was chosen for the following experiments. To study the functional relevance of FGFR1 modulation, recombinant mouse FGF-2 (25 ng/mL; R&D Systems, Abingdon, UK) was administrated (for 0.5 or 48 h) in control and calcitriol-treated HL-1 cells. In addition, suberanilohydroxamic acid (SAHA, 1 μM; Cayman Chemical, Ann Arbor, MI), a potent HDAC inhibitor, was also used to treat HL-1 cells in the presence and absence of calcitriol (10 nM) incubation for 48 h.

### Western blot analysis

HL-1 cells were homogenized and lysed in radioimmunoprecipitation assay buffer containing 50 mM Tris at pH 7.4, 50 mM NaCl, 1% NP40, 0.5% sodium deoxycholate, 0.1% sodium dodecylsulfate (SDS), and a protease inhibitor cocktail (Sigma, St. Louis, MO). Protein concentrations were determined using a Bio-Rad protein assay reagent (Bio-Rad, Hercules, CA). Proteins were subjected to SDS-polyacrylamide gel electrophoresis (PAGE) under reducing conditions and electrophoretically transferred onto equilibrated polyvinylidene difluoride membranes (Amersham Biosciences, Buckinghamshire, UK), as described in a previous study [22]. Blots were probed with primary antibodies against FGFR1 (Cell Signaling Technology, Danvers, MA), FGFR2 (Santa Cruz Biotechnology, Santa Cruz, CA), FGFR3 (Santa Cruz Biotechnology), FGFR4 (Santa Cruz Biotechnology), phosphorylated (p)-ERK (Cell Signaling), p-phospholipase Cγ (PLCγ) (Cell Signaling), nuclear factor of activated T cells (NFAT) (Santa Cruz Biotechnology), and lamin B (Santa Cruz Biotechnology). Secondary antibodies were conjugated with horseradish peroxidase (Leinco Technology, St. Louis, MO). Bound antibodies were detected using an enhanced chemiluminescence detection system (Millipore) and analyzed using AlphaEaseFC software (Alpha Innotech, San Leandro, CA). Targeted bands were normalized to those of glyceraldehyde 3-phosphate dehydrogenase (GAPDH) (Sigma) or lamin B to confirm equal protein loading.

### Real-time reverse-transcription polymerase chain reaction (RT-PCR) analysis

Total RNAs isolated from HL-1 cells were reverse-transcribed using SuperScript III reverse transcriptase (Invitrogen, Carlsbad, CA). FGFR1 and β-myosin heavy chain (β-MHC) messenger (m)RNA expressions were analyzed with a quantitative (q)PCR using the ABI PRISM7300 system (Applied Biosystems, Foster City, CA) and SYBER Green (Applied Biosystems). Relative changes in transcript levels of target genes were estimated from the threshold cycle (Ct) value and normalized to the respective Ct value of GAPDH determined in corresponding samples and subsequently to that of control cells.

### Preparation of cytoplasmic and nuclear extracts from HL-1 cells

Stepwise separation of nuclear and cytoplasmic extracts from HL-1 cells without and with treatment with FGF-2 (25 ng/mL for 30 min) or calcitriol (10 nM for 48 h) was performed using an NE-PER Nuclear and Cytoplasmic Extraction Kit (Thermo Scientific, Waltham, MA). Nuclear and cytoplasmic extracts of control and FGF-2-treated or calcitriol-treated HL-1 cells were subjected to NFAT subcellular localization through a Western blot analysis or HDAC activity measurements, respectively.

### Transfection of small interfering (si)RNA into HL-1 cells for FGFR1 knockdown

HL-1 cells were transfected with 10 nM of either validated mouse FGFR1 siRNA (catalog number: 4390771; Ambion, Austin, TX) or negative control siRNA (catalog number: 4390843) using Lipofectamine RNAiMax Reagent (Thermo Scientific) for 48 h. The protein knockdown efficacy of FGFR1 was assessed 48 h after siRNA transfection. Calcitriol (10 nM) was administered at the beginning of siRNA treatment and incubated for 48 h, whereas FGF-2 (25 ng/mL) was administered for 30 min at 48 h of siRNA transfection (for ERK phosphorylation) or at the beginning of transfection and maintained for 48 h (for β-MHC expression). The sequences of FGFR1 siRNA are as follows: GCUCGAGACAUUCAUCAUATT (sense) and UAUGAUGAAUGUCUCGAGCTA (antisense).

### Measurement of nuclear and cytoplasmic HDAC activities

Respective HDAC activities in the nuclear and cytoplasmic extracts were measured by a fluorometric assay (BioVision, Milpitas, CA) according to the manufacturer’s instructions.

### Chromatin immunoprecipitation (ChIP) assay

The ChIP assay was performed using the Immunoprecipitation Kit Dynal ProteinG (Invitrogen) as per the manufacturer’s instructions. Briefly, chromatin from HL-1 cells treated without and with calcitriol (10 nM) for 48 h was sonicated to a length of 200~ 500 bp. Five micrograms of sheared chromatin was subjected to immunoprecipitation with primary antibodies against acetyl-histone H4 (Millipore, Bedford, MA), HDAC1 (Santa Cruz Biotechnology), HDAC2 (Santa Cruz Biotechnology), HDAC3 (Santa Cruz Biotechnology), and rabbit immunoglobulin G (IgG; Invitrogen). One-tenth of a DNA sample for the ChIP analysis prior to immunoprecipitation was saved for an input control. The input DNA and antibody-bound chromatin were reverse-cross-linked, purified, and then subjected to a PCR using primers designed to amplify a fragment of the FGFR1 promoter containing the vitamin D receptor-binding sites as follows: 5’-TGGGACCAGACTAAAAGCCAAGGGAC-3′, which corresponds to − 1997 to − 1972 nt; and 3’-GGGTAGCCTCAGACTCAAGGACTCTCC-5′, which corresponds to − 1657 to − 1683 nt. Vitamin D receptor-binding sites within the FGFR1 promoter were in silico-identified using ALLGGEN-PROMO software [23]. DNA products after immunoprecipitation with anti-rabbit IgG were used as a negative control. PCR products were then electrophoresed on 2% agarose gels containing DNA View (BioTools, New Taipei City, Taiwan). Band intensities were quantified and normalized to the input control.

### Statistical analysis

All quantitative data are expressed as the mean ± standard error of the mean (SEM). Statistical significance among HL-1 cells under different conditions was determined using a one-way repeated analysis of variance (ANOVA) test with a post-hoc Tukey’s test or paired *t*-test. A *p* value of < 0.05 was considered to indicate a statistically significant difference.

## Results

### Calcitriol decreases FGFR1 expression in HL-1 cells

**Fig. 1:**
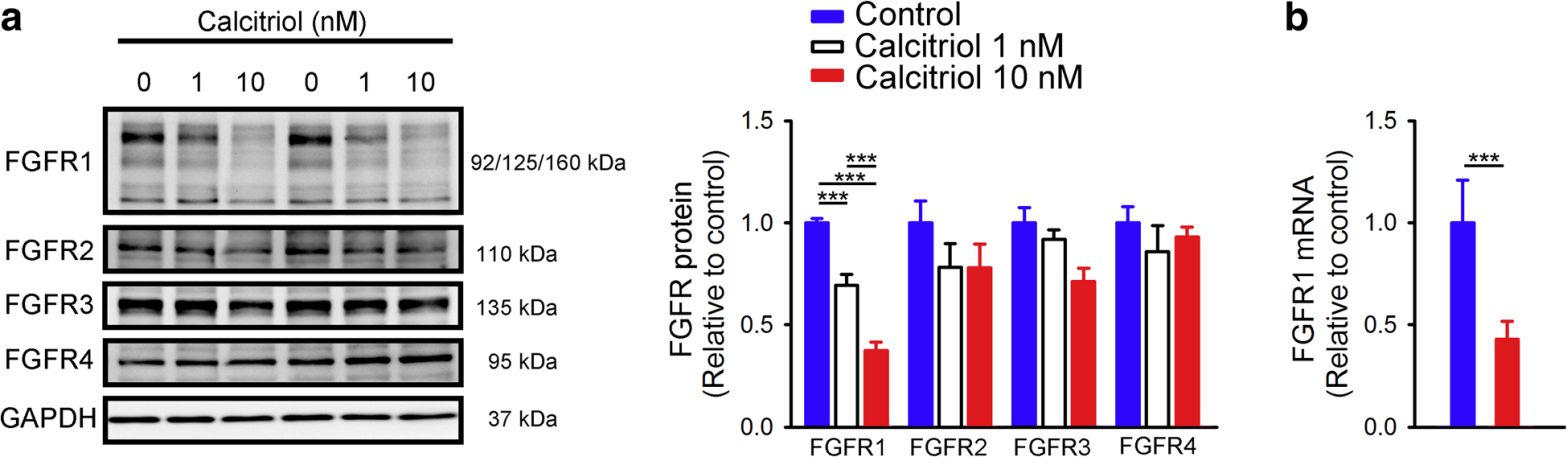
Fibroblast growth factor (FGF) receptor (FGFR) protein and mRNA expressions in control and calcitriol-treated HL-1 cells. (**a**) Representative immunoblots and average data (*n* = 6) of FGFR1, FGFR2, FGFR3, and FGFR4 from control and calcitriol (1 and 10 nM)-treated HL-1 cells, and (**b**) FGFR1 mRNA from control and calcitriol (10 nM)-treated HL-1 cells (*n* = 6). *** *p* < 0.005

Compared to control cells, as shown in Fig. 1a, calcitriol (1 and 10 nM) dose-dependently reduced FGFR1 protein expression in HL-1 cells by 31 and 62%, respectively. In contrast, calcitriol did not significantly change expressions of FGFR2, FGFR3, or FGFR4. Similarly, calcitriol (10 nM)-treated HL-1 cells had lower FGFR1 mRNA expression than did control HL-1 cells (Fig. 1b).

### Calcitriol attenuates FGF-2’s effect on HL-1 cells through FGFR1 inhibition

**Fig. 2:**
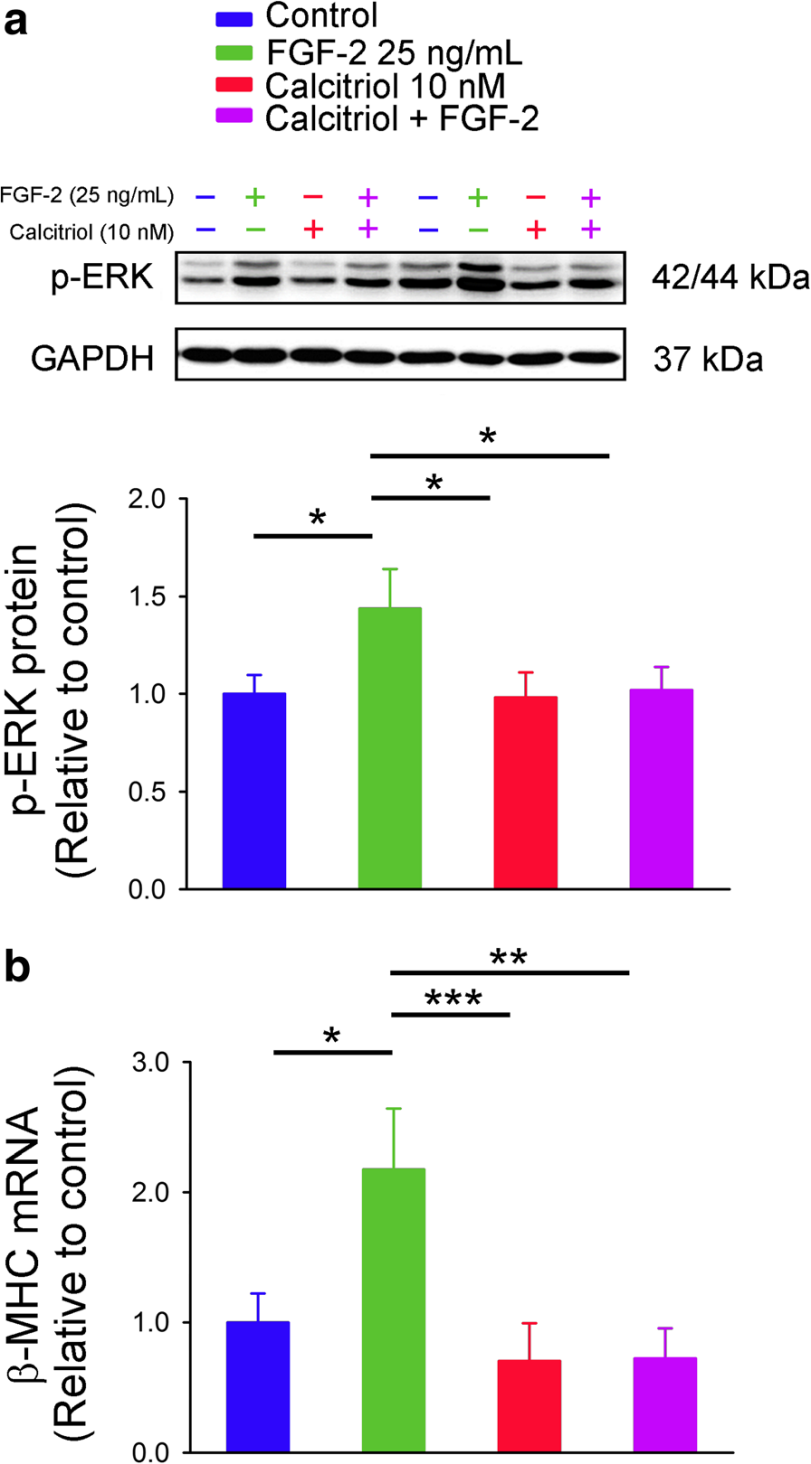
Phosphorylated extracellular signal-regulated kinase (p-ERK) protein and β-myosin heavy chain (β-MHC) mRNA expressions in control and calcitriol-treated HL-1 cells without and with fibroblast growth factor (FGF)-2 incubation. (**a**) FGF-2 (25 ng/mL) was administered for 30 min to study ERK phosphorylation (*n* = 6), or (**b**) for 48 h to evaluate β-MHC mRNA from control and calcitriol (10 nM)-treated HL-1 cells (*n* = 6). * *p* < 0.05, ** *p* < 0.01, *** *p* < 0.005

In order to study whether calcitriol-induced FGFR1 downregulation can modulate the effects of FGF-2, we analyzed phosphorylation of ERK (the downstream signaling pathway of FGFR1 activation) in FGF-2-treated HL-1 cells without and with the co-administration of calcitriol (10 nM). We found that FGF-2 (25 ng/mL for 30 min)-treated HL-1 cells had higher p-ERK expression than that of control or calcitriol (10 nM)-treated cells (Fig. 2a). In contrast, control cells and HL-1 cells treated with FGF-2 (25 ng/mL) combined with calcitriol (10 nM) had similar p-ERK expressions. Moreover, we also compared the expression of β-MHC, which is upregulated in cardiac hypertrophy, and found that FGF-2 (25 ng/mL for 48 h)-induced β-MHC mRNA upregulation was significantly attenuated in calcitriol (10 nM)-treated HL-1 cells (Fig. 2b).

**Fig. 3:**
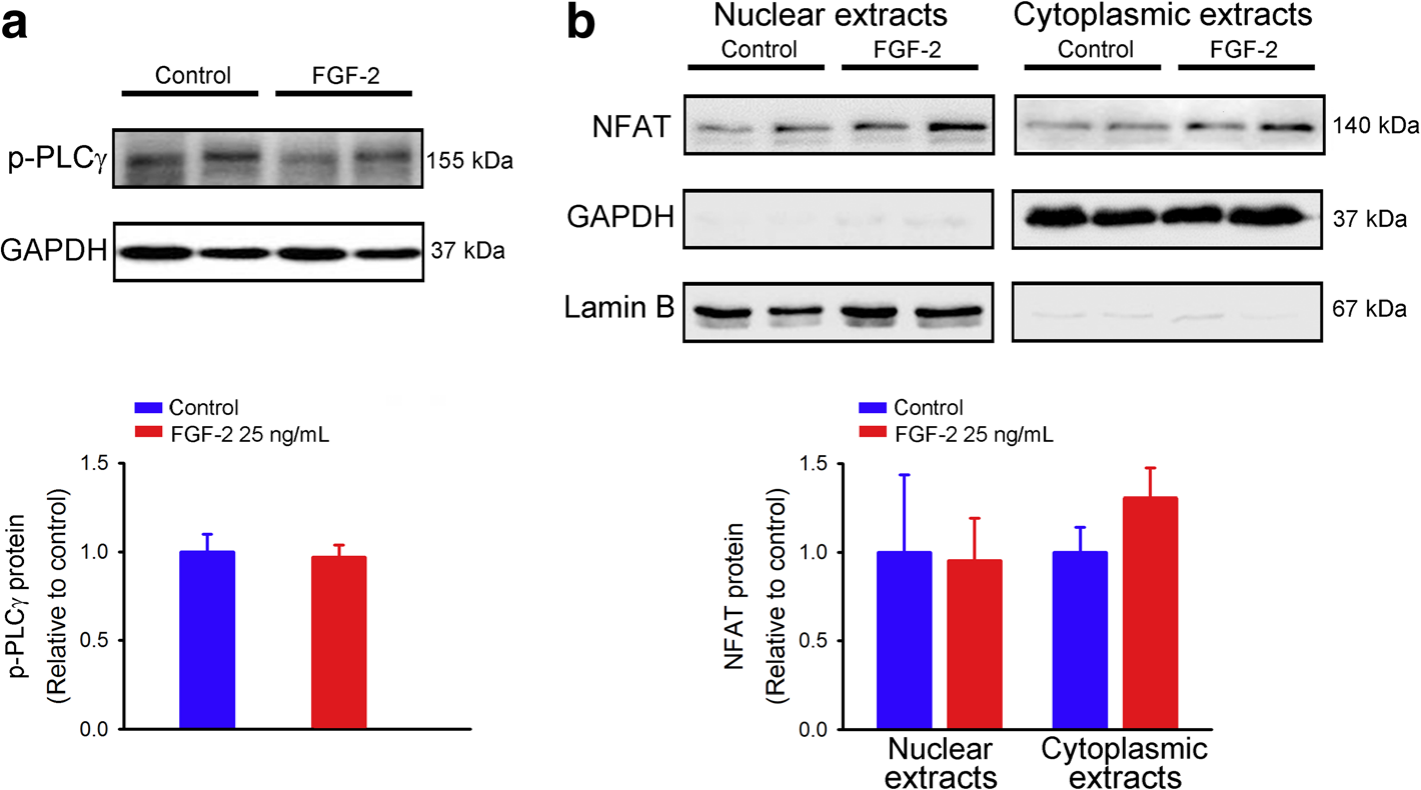
Phosphorylated phospholipase Cγ (p-PLCγ) and nuclear factor of activated T cells (NFAT) protein expressions in control and fibroblast growth factor (FGF)-2-treated HL-1 cells. (**a**) Representative immunoblots and average data (*n* = 6) of p-PLCγ in total cell lysates, and (**b**) NFAT in nuclear and cytoplasmic extracts from HL-1 cells without and with FGF-2 (25 ng/mL) treatment for 30 min

We also investigated the effect of FGF-2 on the PLCγ/NFAT pathway in HL-1 cells and found that FGF-2 (25 ng/mL for 30 min)-treated HL-1 cells had similar levels of PLCγ phosphorylation compared to control cells (Fig. 3a). Additionally, control and FGF-2 (25 ng/mL for 30 min)-treated HL-1 cells had similar expressions of nuclear and cytoplasmic NFAT, suggesting that FGF-2 did not activate PLCγ/NFAT signaling in HL-1 cardiomyocytes (Fig. 3b).

**Fig. 4:**
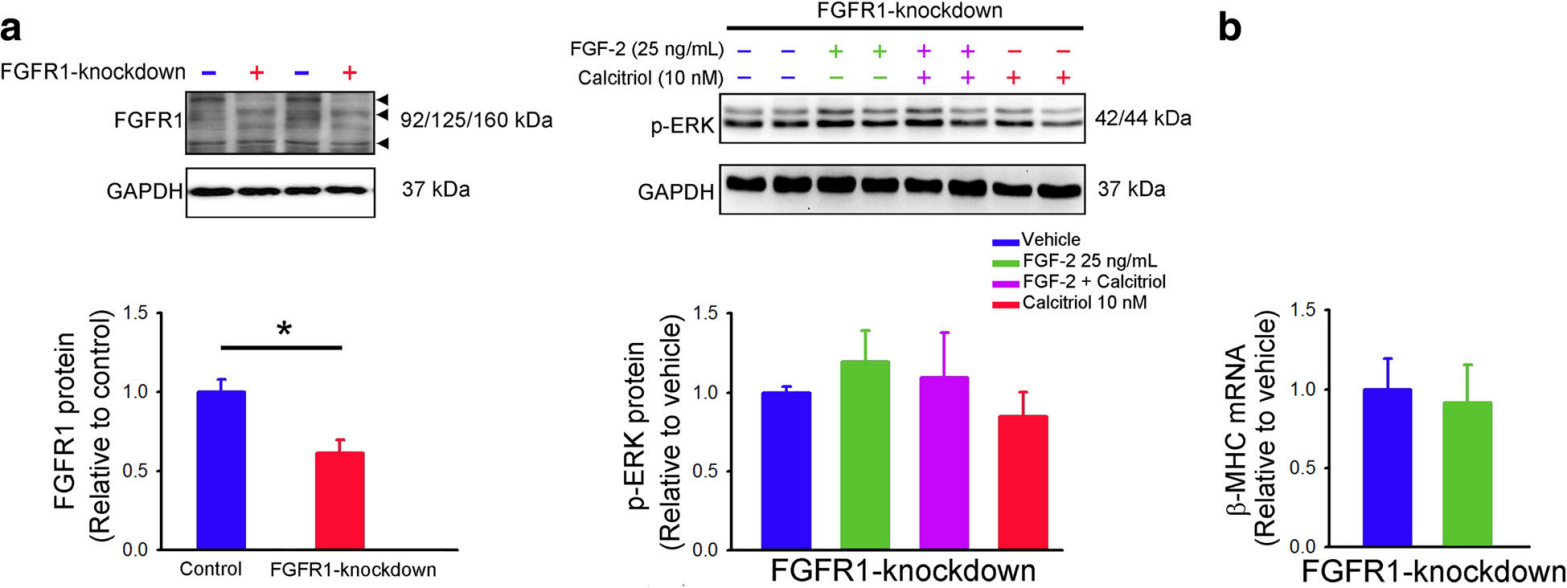
Effects of fibroblast growth factor (FGF) receptor 1 (FGFR1) knockdown on phosphorylated extracellular signal-regulated kinase (p-ERK) protein, and β-myosin heavy chain (β-MHC) mRNA expressions in HL-1 cells. (**a**) Representative immunoblots and average data (*n* = 6) of FGFR1 from control and FGFR1-knockdown cells (left panel). FGF-2 (25 ng/mL for 30 min) did not increase ERK phosphorylation in FGFR1-knockdown cells without and with calcitriol (10 nM) treatment (right panel). (**b**) FGF-2 (25 ng/mL for 48 h) did not increase β-MHC mRNA expression in FGFR1-knockdown cells (*n* = 6). * *p* < 0.05

We performed FGFR1 silencing to prove that the effects of calcitriol observed are dependent on FGFR1. FGFR1-knockdown cells had significantly decreased FGFR1 protein expression than control cells (Fig. 4a). FGF-2 (25 ng/mL for 30 min) did not increase p-ERK expression in FGFR1-knockdown cells. When FGFR1 was knocked down, there were similar expressions of p-ERK among FGF-2 (25 ng/mL for 30 min)-treated, FGF-2 (25 ng/mL for 30 min) combined with calcitriol (10 nM)-treated, or calcitriol (10 nM)-treated cells, suggesting that knockdown of FGFR1 blocked FGF-2 signaling and reduced the protective effects of calcitriol against FGF-2-induced ERK activation. As shown in Fig. 4b, FGFR1-knockdown and FGF-2 (25 ng/mL for 48 h)-treated FGFR1-knockdown cells had similar expressions of β-MHC mRNA. These results suggested that calcitriol attenuated the effects of FGF-2 on HL-1 cells through FGFR1 inhibition.

### Calcitriol reduces FGFR1 expression through HDAC3 recruitment in HL-1 cells

**Fig. 5:**
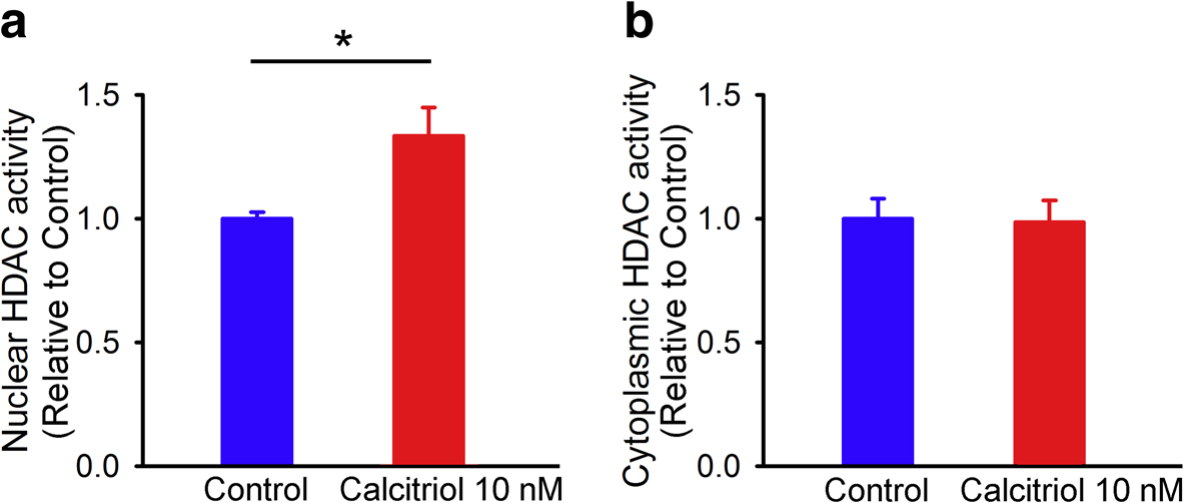
Nuclear and cytoplasmic histone deacetylase (HDAC) activities in control and calcitriol-treated HL-1 cells. Representative average data (*n* = 7) of HDAC activities in (**a**) nuclear and (**b**) cytoplasmic extracts from HL-1 cells without and with calcitriol (10 nM) treatment for 48 h. * *p* < 0.05

Calcitriol (10 nM)-treated HL-1 cells had significantly higher HDAC activity in the nuclear extract than did control cells (Fig. 5a). However, the HDAC activity in the cytoplasmic extract was similar between calcitriol (10 nM)-treated HL-1 cells and control cells (Fig. 5b).

**Fig. 6:**
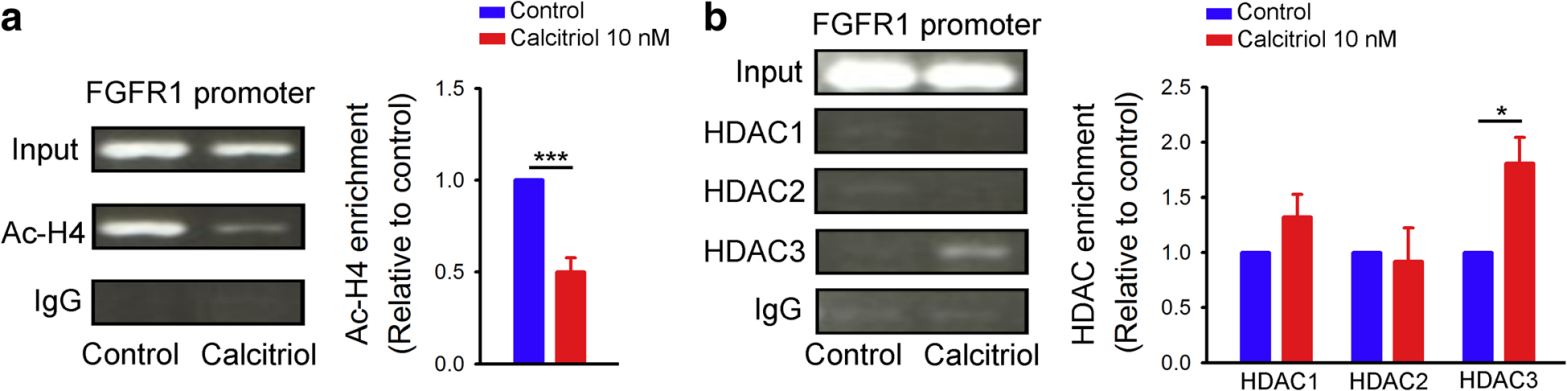
Chromatin immunoprecipitation (ChIP) assay of acetyl-histone H4 (Ac-H4) and histone deacetylase (HDAC) in the fibroblast growth factor receptor 1 (FGFR1) promoter in control and calcitriol-treated HL-1 cells. Representative image and average data (*n* = 6) of (**a**) histone H4 acetylation, and (**b**) HDAC1, HDAC2, and HDAC3 binding to the FGFR1 promoter from control and calcitriol (10 nM)-treated HL-1 cells. * *p* < 0.05, *** *p* < 0.005

**Fig. 7:**
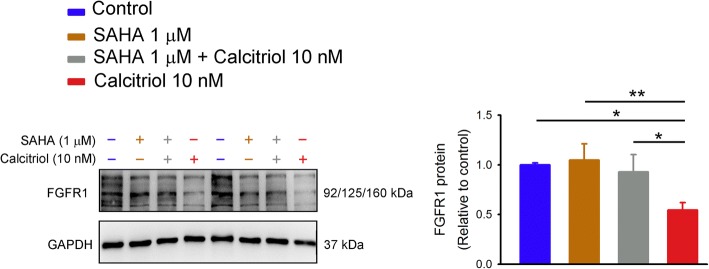
Fibroblast growth factor receptor 1 (FGFR1) protein expression in control and calcitriol-treated HL-1 cells without and with suberanilohydroxamic acid (SAHA, a pan-HDAC inhibitor) treatment. Representative immunoblots and average data (*n* = 7) of FGFR1 from control and calcitriol (10 nM)-treated HL-1 cells without and with SAHA (1 μM) treatment. * *p* < 0.05, ** *p* < 0.01

We examined the acetylation status of the FGFR1 promoter to verify the functional consequences of HDAC activation in calcitriol (10 nM)-treated HL-1 cells. The ChIP analysis showed a significant decrease in acetyl-histone H4 in the FGFR1 promoter in the presence of calcitriol (10 nM) (Fig. 6a). In addition, there was a greater amount of HDAC3 recruited to the FGFR1 promoter in calcitriol (10 nM)-treated HL-1 cells than in control cells (Fig. 6b). Calcitriol did not enhance HDAC1 or HDAC2 recruitment in the FGFR1 promoter in HL-1 cells. We used SAHA, a pan-HDAC inhibitor, to block HDAC activity in studied cells and found that HL-1 cells treated with SAHA (1 μM) combined with calcitriol (10 nM) had significantly higher FGFR1 expression than did calcitriol (10 nM)-treated HL-1 cells, which suggests that activation of HDAC by calcitriol significantly contributed to the effects of calcitriol on FGFR1 downregulation (Fig. 7).

## Discussion

In this study, for the first time, we found that calcitriol downregulated FGFR1 expression in cardiac myocytes. Calcitriol diminished increased p-ERK and β-MHC expressions in FGF-2-treated HL-1 cells. These findings suggest that calcitriol may attenuate the deleterious effects of FGF-2 on the heart by suppressing FGFR1 expression. In addition, the calcitriol-induced FGFR1 reduction was mediated by HDAC3 activation.

The potential of FGF-2 to induce cardiac hypertrophy was first inferred from in vitro studies in which FGF-2 provoked fetal contractile protein gene expression, including upregulation of β-MHC and α-skeletal actin in association with downregulation of α-MHC, which is characteristic of pressure-overload hypertrophy in cultured neonatal rat cardiac myocytes [24, 25]. Kaye et al. demonstrated that exogenous FGF-2 increased protein synthesis and myocyte sizes in isolated adult rat ventricular myocytes [26]. The hypertrophic effect of human pericardial fluid, which was collected from patients undergoing cardiac surgery, was, at least in part, due to its high FGF-2 content [25, 27]. In addition, a previous study found no differences in heart weight-to-body weight ratios or echocardiographic morphometric and functional measurements between wild-type mice and mice with cardiac-specific overexpression or deletion of FGF-2, implying no spontaneous cardiac hypertrophy as a result of manipulation of endogenous FGF-2 expression [4]. However, FGF-2 is essential for the development of pressure overload- or angiotensin II-induced cardiac hypertrophy [3–5]. Therefore, regulating FGF-2 signaling holds promise for treating CVDs.

In mammals, FGFRs constitute a family of four distinct isoforms (FGFR1~ 4) with different FGF-binding specificities. Substantial evidence indicates that FGFR1 is required for the biological activities of FGF-2 [1, 2]. Our study showed that calcitriol significantly reduced FGFR1 protein expression in HL-1 cells. In contrast, calcitriol did not alter expressions of FGFR2, FGFR3, or FGFR4, suggesting that the effects of calcitriol may be relatively specific to FGFR1. Binding of FGF-2 to FGFR1 induces autophosphorylation of FGFR1, thereby activating several signaling pathways including the rat sarcoma protein (RAS)-mitogen-activated protein kinase (MAPK), phosphoinositide 3-kinase (PI3K)-AKT serine/threonine kinase (AKT), and PLCγ pathways [1, 2, 7]. ERK signaling acts as a major regulator of cardiac hypertrophy and myocyte survival. Activated ERK is translocated to nuclei where it phosphorylates transcription factors linked to the hypertrophic response [25]. It was demonstrated that FGF-2 (25 ng/mL) significantly increases cell surface area of neonatal rat ventricular cardiomyocytes. ERK inhibition completely prevents FGF-2-induced hypertrophy [28]. Our findings revealed that calcitriol alleviated FGF-2-induced ERK phosphorylation and β-MHC upregulation in HL-1 cells. PLCγ/NFAT signaling has been shown to be critically involved in cardiac hypertrophy [25]. However, our findings showed that FGF-2 did not activate the PLCγ/NFAT pathway in HL-1 myocytes. Similarly, FGF-2 has been shown to have no effect on PLCγ phosphorylation in HEK293 cells through immunoprecipitation and Western blot analyses. A chemiluminescence reporter assay revealed that FGF-2 did not increase the transcriptional activity of NFAT in neonatal rat ventricular myocytes [8]. Previous studies showed that FGFR blockade prevented development of left ventricular hypertrophy and ameliorated established left ventricular hypertrophy in a rat model of chronic kidney disease [28, 29]. Accumulating evidence suggests that vitamin D plays a critical role in cardiovascular homeostasis, and laboratory findings indicated that calcitriol may be a potential treatment for CVDs [12]. We found that FGFR1-knockdown diminished the prohypertrophic effects of FGF-2, and attenuated the protective effects of calcitriol, suggesting that the effects of calcitriol observed in this study are dependent on FGFR1. Although our setting only produced modest levels of FGFR1 knockdown, this effect abolished the FGF-2-mdiated ERK phosphorylation, suggesting that FGFR1 is critical for FGF-2-activated ERK signaling in our in vitro model. Therefore, repression of FGFR1 by calcitriol has functional significance, and may modulate the hypertrophic effect of FGF-2 on cardiac myocytes.

HDACs are grouped into four classes based on their functions and similarities of their DNA sequences. Class I HDACs, consisting of HDAC1, − 2, − 3, and − 8 are primarily found in nuclei [30, 31]. A previous study demonstrated that calcitriol inhibited IgE production by means of HDAC1- and − 3-mediated transrepressive activity [32]. Calcitriol was shown to ameliorate autoimmunity and exhibit transcriptional repression of the parathyroid hormone through recruitment of HDAC2 [33, 34]. In addition, calcitriol regulates insulin-like growth factor-binding protein 3 gene transcription through cyclical association of the vitamin D receptor with HDAC4 and − 6 [35]. In this study, we found that calcitriol enhanced nuclear HDAC activity in HL-1 cells. The ChIP analysis confirmed the decreased acetylation of histone H4 in the FGFR1 promoter in calcitriol-treated HL-1 cells. Moreover, the suppressive effect of calcitriol on FGFR1 expression was diminished in the presence of an HDAC inhibitor. These findings suggest that HDAC activation is involved in the regulatory mechanism of calcitriol-mediated FGFR1 downregulation. Our study also revealed that HDAC3 was recruited to the FGFR1 promoter in cardiomyocytes following calcitriol treatment.

Our study had some limitations. This study showed that calcitriol may modulate FGFR1 expression in HL-1 cells. HL-1 cells were suggested to retain phenotypic characteristics of adult cardiomyocytes. Multiple investigations using microscopic, genetic, IHC, electrophysiological, and pharmacological techniques demonstrated that HL-1 cells are similar to primary cardiomyocytes [21, 36]. Nevertheless, HL-1 cells are relatively immature due to their proliferative potential. It is not clear whether our findings can be completely applied to in vivo settings. In addition, the concentration of calcitriol used in this study was supra-physiological. It was shown that the serum calcitriol concentration may be as high as around 10 nM in a clinical trial with a high dose of calcitriol for cancer treatment [37]. Since calcitriol is expected to be more concentrated at the cellular level due to its lipophilic property, we studied the effects of calcitriol at 10 nM. However, calcitriol at different concentrations may have different biological activities [38]. In this study, FGFR1 knockdown was confirmed 48 h after FGFR1 siRNA transfection, which may not fully correlate with the activities of FGFR1 following calcitriol or FGF-2 treatment because the efficacy of knockdown may be transient sometimes. Moreover, the molecular mechanism underlying calcitriol-mediated HDAC recruitment remains to be elucidated. It is uncertain whether the effect of calcitriol is being mediated specifically via ERK signaling because we did not manipulate p-ERK activity in this study.

## Conclusions

Calcitriol reduced cardiac FGFR1 expression through HDAC activation. The suppressive effect of calcitriol on FGFR1 was correlated with its reversal of FGF-2-induced harmful effects on cardiac myocytes.

## Abbreviations

ChIP: Chromatin immunoprecipitation
Ct: Threshold cycle
CVDs: Cardiovascular diseases
FGF: Fibroblast growth factor
FGFR: Fibroblast growth factor receptor
GAPDH: Glyceraldehyde 3-phosphate dehydrogenase
HDAC: Histone deacetylase
IHC: Immunohistochemical
PAGE: Polyacrylamide gel electrophoresis
PCR: Polymerase chain reaction
SAHA: Suberanilohydroxamic acid
SDS: Sodium dodecylsulfate
